# Supplementary material for: The Human Meconium Metabolome and Its Evolution during the First Days of Life
Source: Metabolites. 2022 May 5;12(5):414. doi: 10.3390/metabo12050414 (PMC9147484; doi:10.3390/metabo12050414)
Supplement: Supplementary file 1 [file metabolites-12-00414-s001.zip › Figure S1.pdf]

Supplementary Materials:

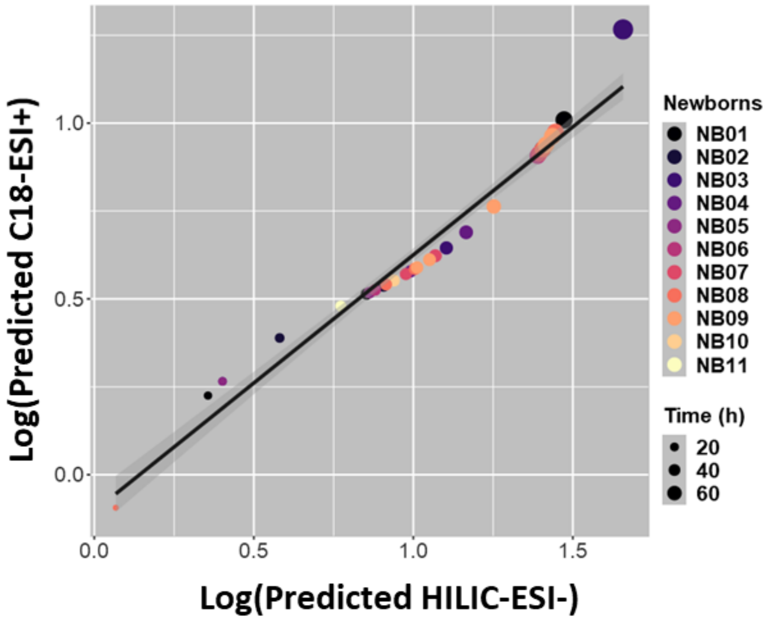

Figure S1. Linear variation of log(Predicted-C18-ESI^{+}) data according to log(Predicted-HILIC-ESI^{-}) data established from the respective modelled Comp[1] scores according polynomials with respective degrees 3 and 4 (see ***Error! Reference source not found.****). Samples collected for the different newborns are indicated in different colors and time-points are indicated according the size of points.*

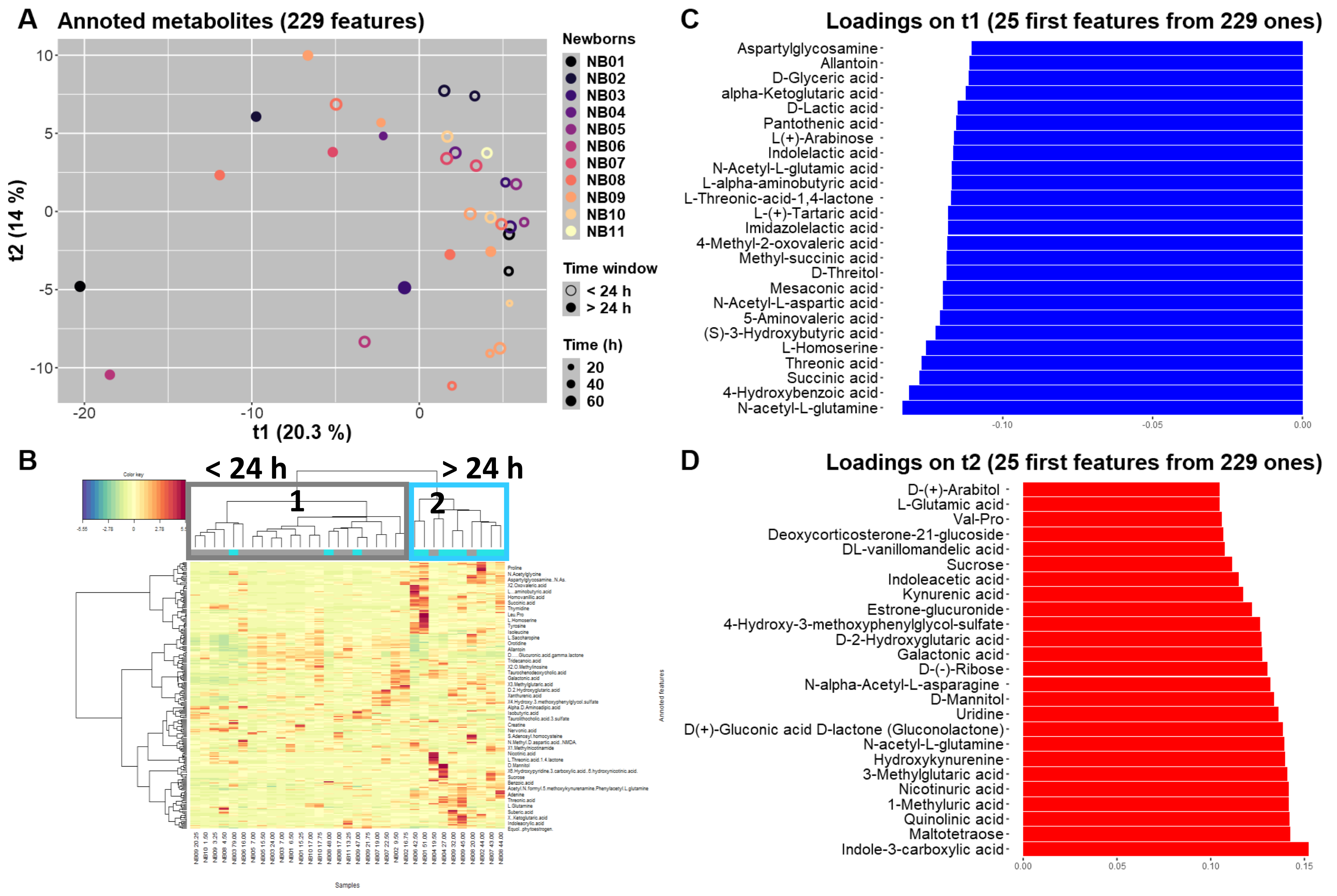

Figure S2. A. PCA performed on the dataset built with the 229 metabolites identified in meconium using 10 latent variables. Samples collected for the different newborns are indicated in different colors and time-points are indicated according to the point size. Open symbols are assigned to samples collected in the first 24 hours when points are assigned to samples collected on the second or third day. B. Non-supervised clustering of samples (in columns, n = 33 samples) based on the 229 identified metabolites (in rows). Colors showed the intensity of each feature in each sample, compared to the mean values obtained in all samples. Except three samples, main part of samples collected in the first 24 hours are clustered in cluster 1 (grey rectangle) when main part of samples collected on the second or third day are clustered in cluster 2 (light blue rectangle). C, D. 25 first loadings on the components t1 and t2, respectively, of PCA displayed in A.
